# Molecular Characterization and Functional Analysis of *Amhr2* in Sex Differentiation and Gonadal Development of Blotched Snakehead (*Channa maculata*)

**DOI:** 10.3390/ijms27114884

**Published:** 2026-05-28

**Authors:** Junqi Qin, Yang Zhang, Jiayuan Shi, Qing Luo, Haiyang Liu, Shuzhan Fei, Xincheng Zhang, Yuandong Sun, Jian Zhao, Mi Ou

**Affiliations:** 1 China (Guangxi)-ASEAN Key Laboratory of Comprehensive Exploitation and Utilization of Aquatic Germplasm Resources, Ministry of Agriculture and Rural Affairs, Key Laboratory of Aquaculture Genetic and Breeding and Healthy Aquaculture of Guangxi, Guangxi Academy of Fishery Sciences, Nanning 530021, China; junqiqin@163.com; 2 Key Laboratory of Tropical and Subtropical Fishery Resources Application and Cultivation, Ministry of Agriculture and Rural Affairs, Pearl River Fisheries Research Institute, Chinese Academy of Fishery Sciences, Guangzhou 510380, China; dy211004@163.com (Y.Z.); 19825305074@163.com (J.S.); luoqing@prfri.ac.cn (Q.L.); hyliu@prfri.ac.cn (H.L.); feisz@prfri.ac.cn (S.F.); zhangxc@prfri.ac.cn (X.Z.); 3 School of Life and Health Sciences, Hunan University of Science and Technology, Xiangtan 411201, China; syd@hnust.edu.cn; 4 College of Animal Science and Technology, Yangzhou University, Yangzhou 225000, China

**Keywords:** *Amhr2*, *Channa maculata*, expression profile, estrogen treatment, CRISPR/Cas9

## Abstract

The anti-Müllerian hormone type II receptor (Amhr2) is a critical component of the transforming growth factor-β (TGF-β) signaling pathway and plays essential roles in sex determination and gonadal development in teleosts. However, its function in the blotched snakehead (*Channa maculata*), an economically important fish in China, remains unexplored. In this study, we cloned and characterized the Amhr2 ortholog from *C. maculata*, designated *CmAmhr2*. The gene encodes a 443-amino acid protein containing a conserved STYKc kinase domain. Sequence and phylogenetic analyses revealed that *Cm*Amhr2 is homologous to autosomal Amhr2 in other teleosts. Spatiotemporal expression analyses showed that *CmAmhr2* was predominantly expressed in testes, particularly during critical windows of gonadal differentiation. In situ hybridization localized *CmAmhr2* transcripts mainly in spermatogonia, with weaker signals in primary spermatocytes, Sertoli cells, and early-stage oocytes (oogonia and primary oocytes). Dietary administration of 30 mg/kg 17β-estradiol (E_2_) from 15 to 45 days post-fertilization (dpf) for 30 days induced male-to-female sex reversal, producing neofemales (XY-F) and intersex individuals (XY-I). *CmAmhr2* expression levels progressively declined with the degree of gonadal feminization: highest in normal XY male (XY-M) testes, intermediate in XY-I ovotestes, and lowest in fully feminized XY-F and normal XX female (XX-F) ovaries. Furthermore, CRISPR/Cas9-mediated mutagenesis of *CmAmhr2* generated frameshift mutations predicted to disrupt the kinase domain. These findings suggest that *CmAmhr2* is involved in male sex differentiation and testis development in *C. maculata*, providing novel molecular insights and a foundation for future sex-control research in aquaculture.

## 1. Introduction

Unlike the relatively conserved genetic sex determination (GSD) systems in mammals, fish exhibit remarkable diversity, ranging from strict GSD to environmental sex determination (ESD) influenced by temperature, pH, or exogenous hormones, often through complex genotype–environment interactions [1]. This plasticity positions fish as valuable models for investigating gonadal fate regulation. Sex determination is initiated by a signaling cascade triggered by sex-determining (SD) genes on sex chromosomes, directing bipotential gonads toward either ovarian or testicular development [2]. Among the key pathways, the transforming growth factor-β (TGF-β) superfamily, particularly Anti-Müllerian hormone (Amh) and its cognate type II receptor (Amhr2), constitutes a key regulatory module for testicular development across teleosts [3].

The Amh/Amhr2 pathway is evolutionarily conserved in vertebrate sex differentiation but has undergone functional diversification. In mammals, Amh drives Müllerian duct regression, a process essential for male reproductive tract development [4]. As the dedicated type II receptor, Amhr2 binds Amh, recruits and activates a type I receptor, and transduces signals via Smad proteins to modulate downstream gene expression [5]. Although teleosts lack Müllerian ducts, the elevated gonadal expression of both *Amh* and *Amhr2* suggests the co-option of this pathway for lineage-specific functions [3]. For instance, *Amhr2* functions as an SD gene in several pufferfish (*Takifugu rubripes*, *T. pardalis*, and *T. poecilonotus*) [6,7,8]. Conversely, certain cyprinids, including zebrafish (*Danio rerio*), grass carp (*Ctenopharyngodon idella*), common carp (*Cyprinus carpio*), and bluntnose black bream (*Megalobrama amblycephala*), have lost *Amhr2* during evolution, with its function possibly taken over by another type II receptor such as bone morphogenetic protein receptor type 2 (*Bmpr2*) [9]. In contrast, a Y-chromosome duplicate, *Amhr2y*, has emerged as a master male-determining gene in a wide range of species, including yellow perch (*Perca flavescens*) [10], Ayu (*Plecoglossus altivelis*) [11], common seadragon (*Phyllopteryx taeniolatus*), alligator pipefish (*Syngnathoides biaculeatus*) [12], southern catfish (*Silurus meridionalis*) [13], Pangasiidae catfish [14], Amur catfish (*S. asotus*) [15], Midas cichlids [16], and spotted knifejaw (*Oplegnathus punctatus*) [17]. These observations underscore the remarkable diversity and evolutionary flexibility of sex determination pathways in fish.

Functional studies using CRISPR/Cas9 gene editing have further elucidated the roles of this pathway. In medaka (*Oryzias latipes*), *Amhr2* mutation (the *hotei* mutant) leads to excessive germ cell proliferation and impaired sex differentiation [18]. In Nile tilapia (*Oreochromis niloticus*), the Y-chromosome Amhy functions as a master SD gene; the disruption of either *Amhy* or *Amhr2* causes complete male-to-female sex reversal in XY individuals [19]. Likewise, in Japanese flounder (*Paralichthys olivaceus*), loss of *Amhr2* results in gonadal reversal in XY males, with evidence suggesting that the pathway promotes masculinization through suppression of estrogen synthesis [20]. Collectively, these findings establish the Amh/Amhr2 signaling pathway as a central regulator of male sex determination and differentiation across diverse fish species, despite substantial variation in genomic configuration and precise roles among teleost lineages.

Blotched snakehead (*Channa maculata*) is an economically important fish in China, valued for its excellent meat quality, high nutritional content, and few intermuscular bones. In 2024, its annual production reached 595,498 tons [21]. It also serves as a valuable model for studying sex determination and sexual dimorphism, as males exhibit consistently superior growth rates and larger body size than females [22]. Previous genetic analyses have confirmed an XX/XY sex-determination system in *C. maculata*, with LG2 identified as the sex chromosome [22]. However, the underlying mechanisms of sex determination and differentiation in this species remain unclear. In this study, to investigate the molecular cascade of male gonadal differentiation in *C. maculata*, we cloned *Amhr2* gene in *C. maculata* (designated *CmAmhr2*), a key regulator of germ cell differentiation and gonadal development, analyzed its expression patterns in different adult tissues and across gonadal development stages, examined its response to exogenous 17β-estradiol (E_2_) treatment, and generated mosaic P0 mutants using CRISPR/Cas9 for further functional characterization. This work provides fundamental insights into the molecular regulation of sex differentiation and gonadal development in *C. maculata* and establishes a theoretical basis for sex-controlled breeding in this species.

## 2. Results

### 2.1. Identification and Molecular Characterization of CmAmhr2

A 1913 bp cDNA sequence encoding *Amhr2* was obtained from *C. maculata* and designated *CmAmhr2* (GenBank accession No. PZ213752). The sequence contains a 272 bp 5′-UTR, a 1332 bp ORF encoding a 443-amino acid protein, and a 309 bp 3′-UTR (Figure S1). Domain analysis identified a signal peptide (residues 1~21) and a STYKc catalytic domain (residues 140~434) that constitutes the kinase core of AMHR2 (Figure S1).

**Figure 1:**
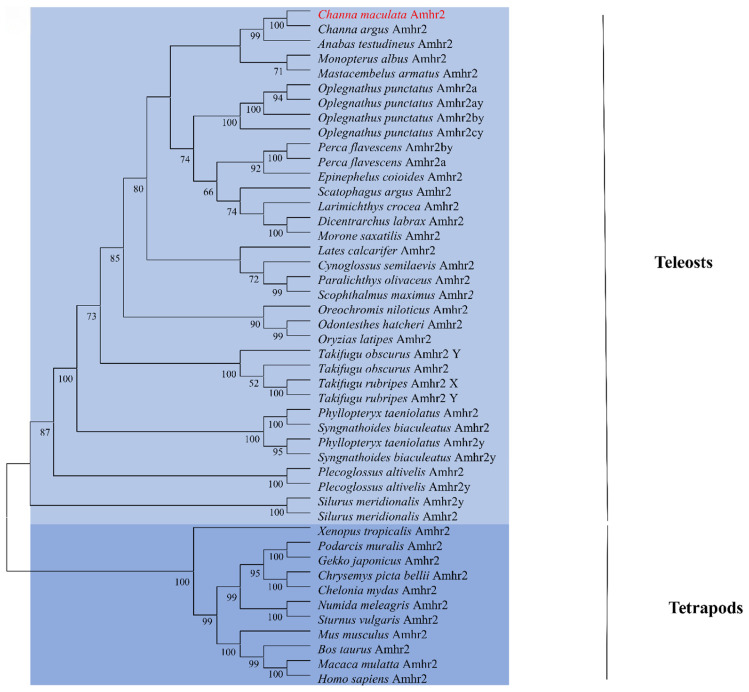
Phylogenetic analysis of Amhr2 amino acid sequences from various vertebrate species. The evolutionary history was inferred using the NJ method. Bootstrap values (1000 replicates) are indicated at branch nodes. Amhr2 from *C. maculata* is highlighted in bold red.

**Figure 2:**
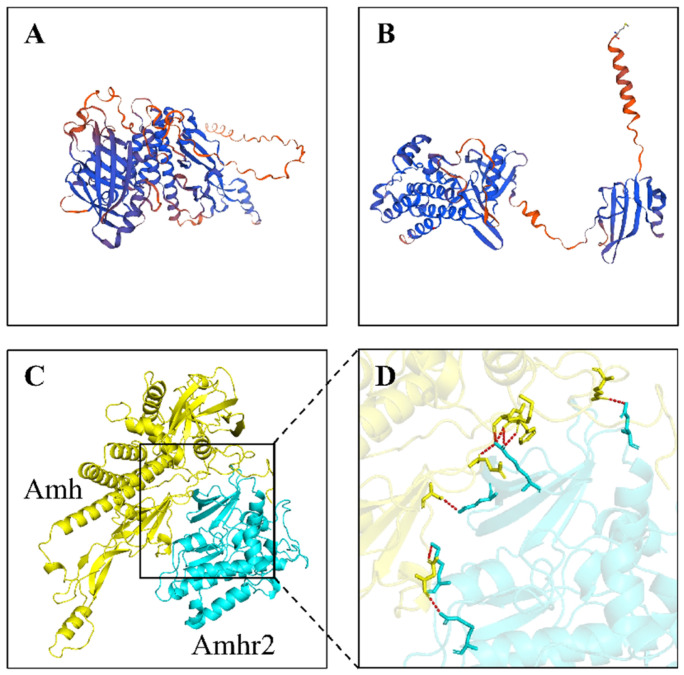
Predicted three-dimensional structures and molecular docking of Amh and Amhr2 from *C. maculata*. (**A**) Amh protein structure. (**B**) Amhr2 protein structure. (**C**) Docking prediction of the Amh-Amhr2 complex. (**D**) Magnified view of the interaction interface from (**C**). In (**A**,**B**), α-helices are shown in orange/red, β-sheets in blue, and random coils in light shades (e.g., pink, purple). In (**C**,**D**), red dashed lines indicate hydrogen bonds between the two protein chains.

*Cm*Amhr2 shared high sequence identity with teleost orthologs (87.58% with *C. argus*, 65.53% with *E. coioides*), but lower identity with bird (*Numida meleagris*, 24.28%) and mammalian (*Mus musculus*, 24.08%) (Figure S2). A neighbor-joining (NJ) phylogenetic tree placed *Cm*Amhr2 within the teleost clade, separate from reptile, amphibian, bird, and mammalian sequences (Figure 1). Predicted three-dimensional structures of *Cm*Amh and *Cm*Amhr2 via SWISS-MODEL revealed characteristic α-helices, β-sheets, and random coils, and molecular docking further revealed a strong predicted interaction between the two proteins (Figure 2). These results confirm that the cloned sequence represents the authentic *Amhr2* ortholog in *C. maculata*.

### 2.2. Genomic Structure Analysis of CmAmhr2

**Figure 3:**
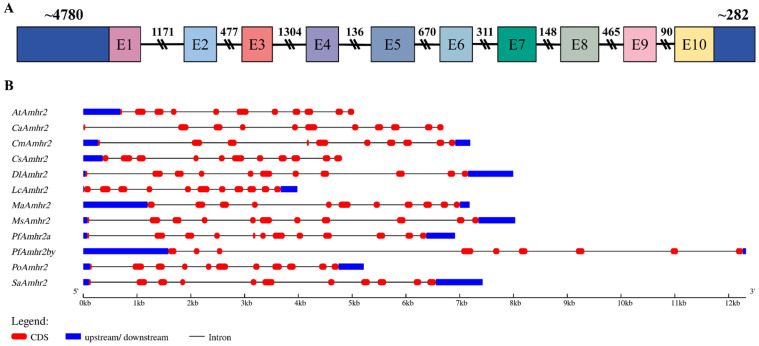
Genomic structure analysis of *Amhr2* orthologs from *C. maculata* and other selected teleosts. (**A**) Schematic diagram of the *CmAmhr2* genomic sequence. Colored boxes represent exons, numbers between boxes indicate intron lengths (in base pairs). (**B**) Comparative structural organization of the *Amhr2* gene across different fish species. Exons are depicted as red rectangles, UTRs as blue rectangles, and introns as connecting lines. Species abbreviations: *At* (*A. testudineus*), *Ca* (*C. argus*), *Cm* (*C. maculata*), *Cs* (*C. semilaevis*), *Dl* (*D. labrax*), *Lc* (*Lates calcarifer*), *Ma* (*Monopterus albus*), *Ms* (*Morone saxatilis*), *Pf* (*P. flavescens*), *Po* (*P. olivaceus*), *Sa* (*Scatophagus argus*).

The full-length genomic sequence of *CmAmhr2* spans 11,706 bp and comprises ten exons (E1~E10) of 37, 189, 165, 41, 222, 115, 152, 157, 137, and 117 bp, respectively, interspersed with nine introns of 1171, 477, 1304, 136, 670, 311, 148, 465, and 90 bp. All exon-intron boundaries conformed to the canonical GT-AG splice donor-acceptor rule (Figure 3A). Comparative analysis across teleosts showed that most species possess eleven exons and ten introns, with introns substantially longer than exons. However, *PfAmhr2a* (*Amhr2* from *Perca flavescens*) contains twelve exons (Figure 3B).

**Figure 4:**
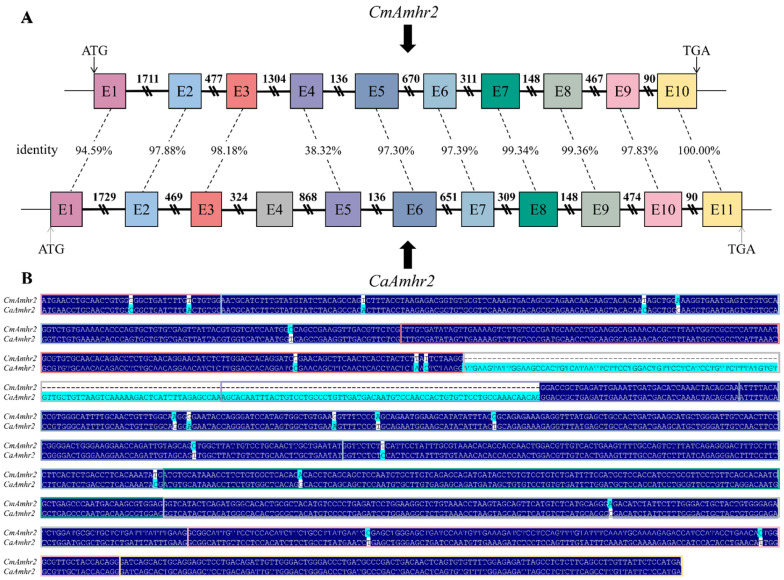
Comparative analysis of the Amhr2 gene structure and coding sequences between *C. maculata* and *C. argus*. (**A**) Schematic representation of the genomic organization of *CmAmhr2* and *CaAmhr2*. Different colored boxes represent distinct exons, numbers between boxes indicate intron lengths (in base pairs). (**B**) Alignment of CDS of *CmAmhr2* and *CaAmhr2*. Different colored boxes indicate different exons. Identical nucleotide residues between the two sequences are highlighted in dark blue.

Comparison with the closely related *C. argus* (*CaAmhr2*) revealed that *CmAmhr2* contains ten exons (E1~E10), whereas *CaAmhr2* has eleven (E1~E11) (Figure 4). Most exons are highly conserved, with identity ranging from 94.59% to 100.00%. The additional exon in *CaAmhr2* (E4) corresponds to the region encoding the transmembrane domain of the AMHR2 protein (Figure S3). Notably, exon 5 (E5) of *CaAmhr2* shows substantially lower identity (38.32%) with exon 4 (E4) of *CmAmhr2*, suggesting potential functional divergence in this region. Subcellular localization predictions showed that *CmAmhr2* was located in the endoplasmic reticulum, whereas *CaAmhr2* was predicted to be a transmembrane protein localized at the cell membrane (Figure S4).

Additionally, a 4780 bp 5′-flanking region and a 282 bp 3′-flanking region of *CmAmhr2* were obtained. Multiple putative TFBS were predicted within the promoter region (−1800~+1), including motifs for AR, Sp1, WT1, DMRT1, Smad2, estrogen receptor (ER), CCAAT/enhancer protein (C/EBP), GATA transcription factor (GATA-1), glucocorticoid receptor (GR), activating protein 1 (AP-1), Sox9, Oct-1, Foxl2, MyoD and Smad3, among others (Figure S5).

### 2.3. Expression Profiles of CmAmhr2 in Tissues and During Gonadal Development

**Figure 5:**
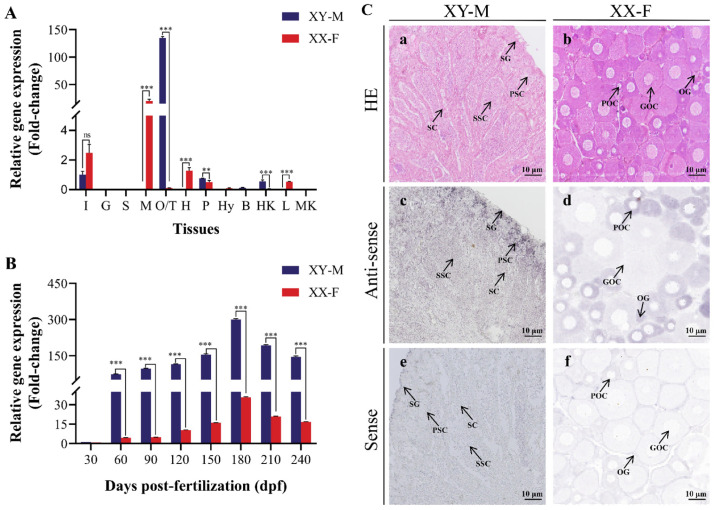
Expression patterns of *CmAmhr2* transcripts. (**A**) Relative expression in different adult tissues, normalized to expression in the intestines (I). (**B**) Relative expression at different developmental stages, normalized to expression at 30 dpf. In (**A**,**B**), *β-actin* was used as the reference gene. Data are mean ± SD (*n* = 3). “ns”: not significant (*p* > 0.05), “**”: *p* < 0.01; “***”: *p* < 0.001. (**C**) Localization of *CmAmhr2* in XY-M testes and XX-F ovaries. (**a**,**b**) HE staining of testes and ovaries, respectively. (**c**,**d**) Location using antisense probes in the testes and ovaries, respectively. (**e**,**f**) Negative controls using sense probes in testes and ovaries, respectively. Positive signals with the antisense probe were indicated in purple. SG: spermatogonia; PSC: primary spermatocyte; SSC: secondary spermatocyte; SC: Sertoli cell; OG: oogonia; POC: primary oocyte; GOC: growing oocyte. Scale bar = 10 µm.

Tissue-specific expression of *CmAmhr2* was examined in XY-M and XX-F individuals using qRT-PCR (Figure 5A). Pronounced sexual dimorphism was observed, most notably in the gonads: expression in XY-M testes reached 134.72 ± 2.48-fold (relative to intestinal baseline), whereas expression in XX-F ovaries was only 0.11 ± 0.01-fold (*p* < 0.001). Moderate, dimorphic expression was also detected in muscle (0.04 ± 0.00-fold in XY-M versus 19.63 ± 3.45-fold in XX-F) and intestines. Low expression levels were found in gills, spleen, hypothalamus, brain, and middle kidney in both sexes.

Expression levels during gonadal development were examined from 30 to 240 dpf (Figure 5B). Transcript levels were minimal and similar between sexes at 30 dpf (1.21 ± 0.02-fold in XY-M testes vs. 1.00 ± 0.01-fold in XX-F ovaries, *p* > 0.05). From 60 dpf onward, expression in XY-M testes progressively increased relative to XX-F ovaries. In XY-M testes, *CmAmhr2* expression increased progressively from 60 dpf (96.51 ± 1.03-fold), peaked at 180 dpf (299.59 ± 3.66-fold), and subsequently declined by 240 dpf (145.27 ± 2.93-fold). In contrast, ovarian expression remained relatively stable throughout development, ranging from 1.00 ± 0.01-fold at 30 dpf to 16.60 ± 0.32-fold at 240 dpf. Between 60~240 dpf, testicular expression was 8.38- to 16.36-fold higher than ovarian expression (*p* < 0.001). Thus, *CmAmhr2* exhibits a male-biased expression pattern that becomes prominent during gonadal differentiation.

### 2.4. Cellular Localization of CmAmhr2 Transcripts in Gonads

Spatial distribution of *CmAmhr2* transcripts was examined by ISH on gonadal sections from 120 dpf XY-M and XX-F individuals (Figure 5C). Adjacent sections stained with HE confirmed the typical gonadal composition. In XY-M testes, spermatogonia (SG), primary spermatocytes (PSC), and secondary spermatocytes (SSC) were clearly distinguishable (Figure 5C-a). XX-F ovaries contained oogonia (OG), primary oocytes (POC), and growing oocytes (GOC) (Figure 5C-b). Upon hybridization with the antisense probe, positive signals in XY-M testes were predominantly localized to SG, with weaker signals in PSC and Sertoli cells (Figure 5C-c). In XX-F ovaries, *CmAmhr2* transcripts were mainly detected in early oocytes, including OG and POC (Figure 5C-d). No specific signals were observed with the sense probe in either testes (Figure 5C-e) or ovaries (Figure 5C-f).

### 2.5. Expression Dynamics of CmAmhr2 Following Exogenous E_2_ Treatment

**Figure 6:**
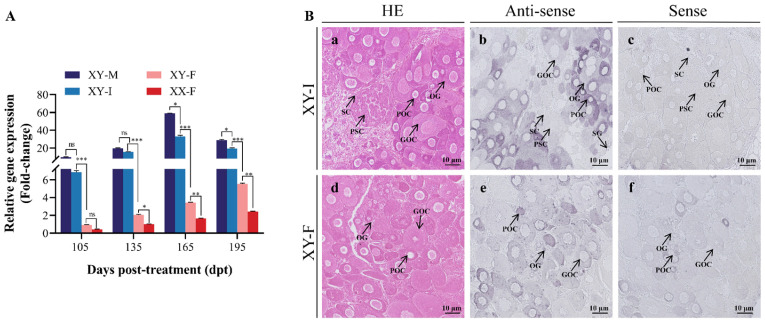
Response of *CmAmhr2* expression to exogenous E_2_. (**A**) Expression levels following E_2_ treatment at different developmental stages, normalized to expression at 105 dpt in XX-F ovaries. “*”: *p* < 0.05, “**”: *p* < 0.01, “***”: *p* < 0.001, ns, not significant. (**B**) Localization of *CmAmhr2* in XY-I ovotestes and XY-F ovaries at 105 dpt. (**a**,**d**) HE staining of XY-I ovotestes and XY-F ovaries, respectively. (**b**,**e**) Location using antisense probes in XY-I ovotestes and XY-F ovaries, respectively. (**c**,**f**) Negative controls using sense probes in XY-I ovotestes and XY-F ovaries, respectively. SG: spermatogonia; PSC: primary spermatocyte; SC: Sertoli cell; OG: oogonia; POC: primary oocytes; GOC: growing oocytes. Scale bar = 10 µm.

The effects of E_2_ treatment on the gonadal development of XY-genotype *C. maculata* were systematically investigated through histological examination and genetic sex identification. At 105 dpt (120 dpf), the sex reversal rate was 64.7%, normal testis, sex-reversed ovaries, and sex-reversed ovotestis were found in the 30 mg/kg E_2_-treated groups (Table 1). Additionally, transcript levels of *CmAmhr2* were assessed in gonads of four phenotypic groups (XY-M, XY-F, XY-I, and XX-F) at 105, 135, 165, and 195 dpt (Figure 6A). Throughout the sampling period, *CmAmhr2* expression remained significantly higher in XY-M testes than in XX-F ovaries (*p* < 0.001). Notably, expression levels declined progressively with the degree of gonadal feminization: highest in XY-M testes, intermediate in XY-I ovotestes, and lowest in fully feminized XY-F and XX-F ovaries. At 105 dpt, expression was highest in XY-M testes (9.73 ± 0.24-fold), followed by XY-I ovotestes (6.84 ± 0.18-fold), with no significant difference between these two groups (*p* > 0.05). In contrast, XY-F and XX-F ovaries showed substantially lower levels (0.92 ± 0.04-fold and 0.44 ± 0.02-fold, respectively). By 135 dpt, expression surged in XY-M testes (19.62 ± 0.47-fold) and XY-I ovotestes (15.69 ± 0.16-fold), while remaining low in XY-F (2.05 ± 0.07-fold) and XX-F ovaries (1.0 ± 0.03-fold). This divergence intensified at 165 dpt, with peak expression in XY-M testes (59.03 ± 0.18-fold) and XY-I ovotestes (33.04 ± 1.40-fold), versus low ovarian levels (XY-F: 3.44 ± 0.03-fold; XX-F: 1.65 ± 0.04-fold). Despite modest declines being noted by 195 dpt (XY-M: 28.67 ± 0.89-fold; XY-I: 19.51 ± 0.65-fold), testicular expression remained significantly higher than ovarian levels (*p* < 0.001) (XY-F: 5.12 ± 0.21-fold; XX-F: 2.34 ± 0.09-fold).

Cellular localization of *CmAmhr2* transcripts following E_2_ treatment was examined by ISH on gonadal sections from 105 dpt individuals (Figure 6B). Adjacent HE-stained sections revealed the cellular composition of each gonadal phenotype. In XY-I ovotestes, both ovarian (OG, POC, and GOC) and testicular (SG, PSC) structures were present (Figure 6B-a), with *CmAmhr2* transcripts detected in early oocytes (OG, POC) and SG (Figure 6B-b). In XY-F individuals, gonads consisted exclusively of ovarian components (OG, POC, and GOC) (Figure 6B-d), with positive signals primarily localized to OG and POC (Figure 6B-e). No specific signals were observed with sense probes in either XY-I (Figure 6B-c) or XY-F (Figure 6B-f) gonads.

### 2.6. Disruption of CmAmhr2 Using CRISPR/Cas9

**Figure 7:**
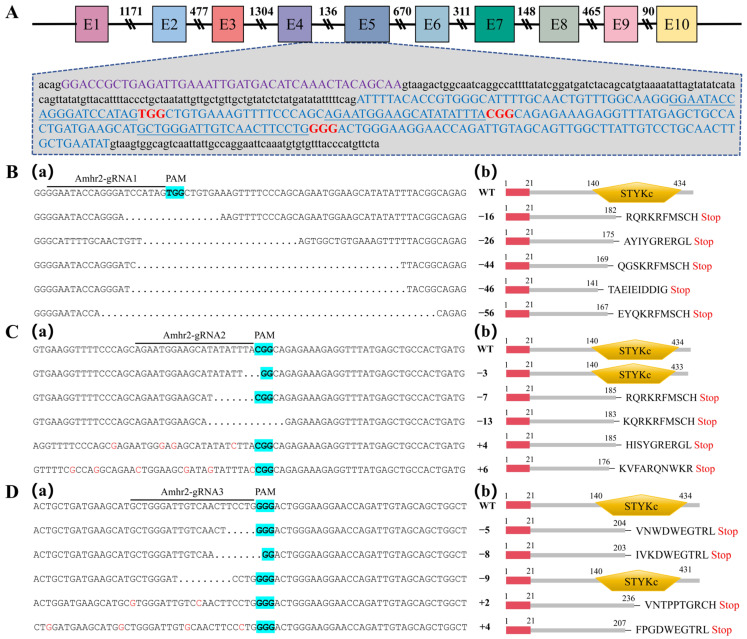
Analysis of *Amhr2* mutation in *C. maculata*. (**A**) CRISPR/Cas9 target sites in the *CmAmhr2* gene. The gRNA target site is underlined, and the PAM is shown in bold red. (**B**–**D**) Mutation types in Amhr2-gRNA1, -gRNA2, and -gRNA3-injected groups, respectively. (**a**) Sequences of *CmAmhr2* showing mutations induced by co-delivered gRNA and Cas9 protein. Wild-type (wt) sequences are shown at the top; the gRNA target site is indicated by an overline. The PAM has a blue background. Deletions are marked by “−, insertions by “+”, and numbers to the right indicate the number of missing or added bases. (**b**) Schematic diagrams showing the predicted intact Amhr2 protein in WT and the truncated Amhr2 protein in mosaic P0 *C.maculata* (see (**a**)). Numbers indicate amino acid residue positions. Frameshift reading produces amino acid sequences after incomplete domains, resulting in a premature termination codon (shown in bold red).

To investigate the functional role of *CmAmhr2* in sex determination and differentiation, CRISPR/Cas9 editing was performed targeting exon 5 of *CmAmhr2*. Three gRNAs were designed adjacent to protospacer adjacent motif (PAM) sequences (Figure 7A). Zygotes at the 1~4 cell stage were microinjected within ~60 min post-fertilization with 1 nL of a solution containing gRNA and Cas9 protein. Fertilization rates were 42.3 ± 3.1%, 41.4 ± 1.4%, 42.7 ± 2.0%, and 41.0 ± 1.2% for the 0.75% NaCl-injected, Amhr2-gRNA1, -gRNA2, and -gRNA3 groups, respectively, with no significant differences among injected groups (*p* > 0.05). All were significantly lower than the untreated control (80.7 ± 2.0%, *p* < 0.05). Similarly, hatching rates were 34.6 ± 1.1%, 34.6 ± 2.0%, 34.2 ± 1.2%, and 33.7 ± 1.5% for the four injected groups, respectively, showing no intergroup differences (*p* > 0.05), but significantly reduced compared to the untreated control (73.1 ± 1.2%, *p* < 0.05). Survival to the flat swimming stage was also markedly affected: from 900 embryos per group, 577 fry survived in the untreated control, whereas only 256, 234, 211, and 219 fry survived in the 0.75% NaCl, Amhr2-gRNA1, -gRNA2, and -gRNA3-injected groups (Table 2, Figure S6), respectively, representing significantly reduced survival relative to untreated control (*p* < 0.05).

Mutation analysis at the target sites was conducted by PCR amplification, subcloning, and sequencing of 48 hpf fry. Mutagenesis frequencies were 50.0%, 43.3%, and 40.0% for the Amhr2-gRNA1, -gRNA2, and -gRNA3-injected groups, respectively (Table 3). Sequencing alignment identified multiple insertions and deletions (indels) induced by the CRISPR/Cas9 system (Figure 7). In the Amhr2-gRNA1 injected group, five deletions (16, 26, 44, 46, and 56 bp) were identified (Figure 7B-a), all non-triplet and predicted to cause frameshifts (Figure 7B-b). In the Amhr2-gRNA2 group, three deletions (3, 7, and 13 bp) and two insertions (4 and 6 bp) were observed (Figure 7C-a). Aside from the 3-bp deletion, all were expected to induce frameshifts (Figure 7C-b). In the Amhr2-gRNA3 group, mutations comprised deletions (5, 8, and 9 bp) and insertions (2 and 4 bp) (Figure 7D-a). The 9-bp triplet deletion led to a three-amino-acid loss without frameshift, whereas all other indels resulted in frameshifts (Figure 7D-b). Given the limited sample size and the mosaic nature of F0 mutants, these mutation frequencies provide only a preliminary indication of genome editing activity, not precise estimates of mutagenesis efficiency. Collectively, these results confirm that all three gRNAs effectively cleaved the genomic *CmAmhr2* locus, with most indels introducing frameshifts and premature termination codons, likely generating truncated, nonfunctional protein products.

## 3. Discussion

As a key member of the TGF-β receptor superfamily, *Amhr2* was identified and characterized in *C. maculata* (designated *CmAmhr2*). The gene encodes a single transcript containing the conserved STYKc domain characteristic of type II TGF-β receptor, consistent with our previous localization of a single *Amhr2* copy on LG05 [23]. Phylogenetic analysis placed *Cm*Amhr2 within the teleost clade, and molecular docking predicted its direct interaction with *Cm*Amh, suggesting a conserved Amh/Amhr2 signaling axis. Recent large-scale surveys indicate that, due to gene duplication or mutation, *Amh* and *Amhr2* homologs serve as SD genes in 34 and 43 teleost species, respectively, accounting for ~50% of reported fish species with identified SD genes [24]. This recurrent recruitment underscores the evolutionary significance of this pathway in teleost sex determination and differentiation, although species-specific variations exist. For instance, many fish, such as *Anabas testudineus* (XM_026347020.1), *Cynoglossus semilaevis* (XM_025060061.1), and *Dicentrarchus labrax* (JQ801443.1), possess a single copy. Notably, comparative genomic analysis revealed that, unlike the *C. argus* ortholog (*Ca*Amhr2), which includes an additional exon encoding the transmembrane domain, *Cm*Amhr2 lacks this feature, consistent with its predicted localization as a soluble protein in the endoplasmic reticulum rather than a membrane-bound receptor. Despite this structural divergence, *Cm*Amhr2 retains the ability to bind *Cm*Amh, implying potential unconventional signaling mechanisms. This finding aligns with evidence of teleost-specific adaptations, such as the use of Bmpr2a as an alternative type II receptor for Amh in *D. rerio* [25]. Such structural variation, possibly arising from lineage-specific events following whole-genome duplication, warrants further functional investigation in *C. maculata*.

Although teleosts lack Müllerian ducts, the Amh/Amhr2 pathway is widely involved in gonadal development, with *Amhr2* predominantly expressed in testes across diverse species, such as largemouth bass (*Micropterus salmoides*) [26], *O. niloticus* [19], *T. rubripes* [6], *P. olivaceus* [20], *E. coioides* [27]. The present study yielded similar results, showing high gonadal expression of *CmAmhr2* with a male bias. However, some cyprinid species, such as *D. rerio*, *C. carpio*, and *C. idella*, have lost *Amhr2*, with its function possibly replaced by another type II receptor, such as *Bmpr2* [9,25]. In certain catfishes, an additional Y-chromosome copy, *Amhr2y*, is testis-specific in *S. meridionalis* [13] and *S. asotus* [15], whereas the autosomal *Amhr2* is expressed in both sexes (higher in testes). Extra-gonadal expression of *Amhr2* has also been reported, including in the head kidney in striped catfish (*Pangasianodon hypophthalmus*) [14], kidney, gill, and spleen in *S. asotus* [15], and kidney, intestine, and heart in male *O. punctatus* [17]. Similarly, *CmAmhr2* exhibited relatively high expression in muscle, and we identified a MyoD transcription factor binding site in its promoter. MyoD, a myogenic regulatory factor specifically expressed in myoblasts and skeletal muscle cells, plays a key role in muscle development and growth [28]. This binding site suggests a potential regulatory link between *CmAmhr2* and muscle growth in *C. maculata*.

During gonadal development, *CmAmhr2* expression was consistently higher in testes than in ovaries, showing an initial increase followed by a decline. Expression was low at 30 dpf (prior to testicular differentiation), increased sharply at 60 dpf (coinciding with the appearance of primary spermatocytes), and peaked at 180 dpf (when testes contained abundant sperm), indicating a close association with testicular differentiation and development in *C. maculata* [25]. Sexually dimorphic expression of *Amhr2* varies among teleosts. In *O. punctatus*, male-biased *Amhr2* expression persists throughout development, with peaks at 6 and 180 days post-hatching (dph) [17]. In contrast, *O. latipes* shows female-biased expression during early development (0~39 dph), switching to male-biased after 60 dph [29]. In *S. asotus* and *S. meridionalis*, the autosomal *Amhr2* is expressed in both sexes but significantly higher in XY gonads at all stages, whereas the Y-linked *Amhr2y* is testis-specific, peaking early and then declining [13,15]. ISH revealed that *CmAmhr2* transcripts in testes were predominantly localized in SG, with weaker signals in PSC and Sertoli cells; in ovaries, low expression was observed in early oocytes. Expression patterns of *Amhr2* vary among teleost species. In *O. niloticus*, *Amhr2* is expressed in SG and Sertoli cells of the testis, as well as in OG, oocytes, and granulosa cells of the ovary [19,30]. In *S. chuatsi*, expression is detected in previtellogenic oocytes and Sertoli cells [31]. In *O. punctatus*, Amhr2 signals are predominantly localized in PSC and SSC, stronger in PSC than in SSC, with weaker signals in the Sertoli cells, and no ovarian signals [17]. Collectively, these findings suggest that *Amhr2* plays a significant role in testis development and may also contribute to ovarian function in certain species.

Exogenous steroid hormones are widely used to induce sex reversal, during which the expression patterns of sex-related genes often change accordingly. In this study, treatment with 30 mg/kg E_2_ successfully induced male-to-female sex reversal in *C. maculata*, accompanied by a significant reduction in *CmAmhr2* expression. Since *Amhr2* is closely associated with testicular differentiation, its downregulation under E_2_ treatment is consistent with the suppression of the male pathway and the promotion of ovarian differentiation. Notably, the extent of *CmAmhr2* suppression appeared to correlate with the degree of feminization observed in gonadal phenotypes, suggesting that reduced *CmAmhr2* expression may contribute to, but not solely determine, sex reversal efficiency. Similar inhibitory effects of E_2_ on testicular *Amhr2* expression have been reported in *Anguilla japonica* [32], *T. rubripes* [33], and black porgy (*Acanthopagrus schlegeli*) [34]. In contrast, in the hermaphroditic *E. coioides*, both *Amh* and *Amhr2* are significantly upregulated during female-to-male sex reversal [27], further supporting the conserved role of the Amh/Amhr2 signaling pathway in male differentiation across teleosts. However, complete sex reversal was not achieved in this study (64.7%), and a proportion of individuals exhibited intersex gonads containing both male (SG and PSC) and female (OG, POC, and GOC) germ cells. This incomplete reversal suggests that while E_2_ treatment effectively suppresses *CmAmhr2*, the degree of suppression may be insufficient in some individuals to fully inhibit testicular development. Several factors may contribute to this variability. First, the administered dose (30 mg/kg) may be too low to induce complete sex reversal in all individuals. Second, uneven hormone intake due to differences in feeding behavior or metabolism could lead to variable internal E_2_ exposure. A similar phenomenon has been reported in *S. chuatsi* [35], where inconsistent hormone uptake resulted in partial sex reversal. Therefore, the occurrence of intersex individuals likely reflects incomplete suppression of the male pathway, highlighting that both the level and consistency of exogenous hormone exposure are critical determinants of sex reversal efficiency. Future studies optimizing dosage, treatment duration, and delivery methods may help achieve more stable and complete sex reversal.

Microinjection is widely used to deliver reagents into early fish embryos but can cause mechanical injury and reduce hatching rates, as reported in several species, such as channel catfish (*Ictalurus punctatus*) [36], sterlet (*Acipenser ruthenus*) [37], *P. hypophthalmus* [38], and large yellow croaker (*Larimichthys crocea*) [39], though not in fathead minnow (Pimephales promelas) [40]. In this study, we microinjected sgRNA/Cas9 complexes into *C. maculata* embryos at the 1~4 cell stage to generate *Amhr2* mutants. Both saline-injected and gRNA/Cas9-injected groups exhibited lower hatching rates than uninjected controls, with no significant difference between the two injected groups, indicating that the injection procedure itself, not the gRNA complexes, reduced hatching. All three designed gRNAs efficiently targeted *CmAmhr2*, with mutagenesis efficiencies of 40~50%. The observed mutations were predominantly frameshifts leading to premature stop codons and disruption of the kinase domain, a region essential for Amhr2 phosphorylation and signaling [5]. Thus, the generated *CmAmhr2* mutants are predicted to exhibit complete loss of Amh/Amhr2 function. Loss-of-function *Amhr2* mutants have been generated in several fish species, including *O. latipes* [18], *O. niloticus* [19], *P. altivelis* [11], *P. olivaceus* [20], and *S. meridionalis* [13]. In *O. latipes* and *O. niloticus*, Amhr2-deficient XY individuals showed male-to-female sex reversal at rates exceeding 50% and 100%, respectively [18,19]. In *P. olivaceus*, loss of *Amhr2* also caused sex reversal, which could be rescued by an aromatase inhibitor, suggesting that the Amh/Amhr2 system influences male differentiation by suppressing estrogen synthesis [20]. Similarly, loss of the Y-linked *Amhr2y* results in complete gonadal sex reversal in XY mutants of *P. altivelis* [11] and *S. meridionalis* [13]. Collectively, these findings underscore the critical role of *Amhr2* in male sex determination and differentiation, gonadal development, and early germ cell regulation in fish. However, because the knockout fish generated in this study are still immature and require two generations to produce homozygous *CmAmhr2*^−/−^ mutants, the effects of *CmAmhr2* deletion on gonadal development and potential sex reversal remain to be determined. Future work will prioritize elucidating the functional role of *CmAmhr2* and the specific molecular mechanisms of its signal transduction.

## 4. Materials and Methods

### 4.1. Experimental Fish and Sampling

Blotched snakeheads were reared at the Fangcun Experiment Station of Pearl River Fisheries Research Institute (Guangzhou, China). Adult XY-M and XX-F were identified using a sex-specific molecular marker and gonadal histology [22]. Tissue samples, including liver (L), gills (G), middle kidney (MK), spleen (S), head kidney (HK), intestines (I), muscle (M), heart (H), gonads (ovaries or testes (O/T)), brain (B), hypothalamus (Hy), and pituitary (P), were collected from one-year-old XX-F and XY-M individuals (*n* = 3 per sex) following anesthesia with tricaine methanesulfonate (MS-222, Sigma-Aldrich, Burlington, MA, USA) at a dose of 1.25 g/mL. These tissues were used for gene cloning and expression analysis.

To investigate *Amhr2* expression during gonadal differentiation, gonads were harvested from randomly selected XX-F and XY-M fish (*n* = 3 per sex per time point) at 30, 60, 90, 120, 150, 180, 210, and 240 dpf. The methods for genetic and physiological sex identification were described above. All samples were immediately frozen in liquid nitrogen and stored at −80 °C for subsequent quantitative real-time PCR (qRT-PCR). Additionally, gonads from 120 dpf XX-F and XY-M individuals were fixed in Bouin’s solution (MedChemExpress, Monmouth Junction, NJ, USA) for hematoxylin and eosin (HE) staining and in situ hybridization (ISH). All experimental procedures were conducted in accordance with animal welfare guidelines and approved by the Animal Ethics Committee of the Pearl River Fisheries Research Institute, Chinese Academy of Fishery Sciences.

### 4.2. Gene Cloning and Sequence Analysis

Total RNA extraction and cDNA synthesis were performed following established protocols [41]. Gene-specific primers (Table 4) for amplifying the *Amhr2* coding sequence were designed based on the blotched snakehead genome (SRA Accession No. PRJNA730430) [42]. The open reading frame (ORF) was amplified from gonadal cDNA via PCR. Products of the expected size were gel-purified using a Gel Rapid Extraction Kit (CWBIO, Taizhou, China), ligated into the pMD18-T vector (Takara, Kusatsu, Japan), and transformed into Trans5α chemically competent cells (TransGen Biotech, Beijing, China). Positive clones were selected and sequenced (Tsingke Biotech, Beijing, China).

Homologous Amhr2 protein sequences from other vertebrates were retrieved from the NCBI database (accession numbers listed in Table 5). Multiple sequence alignment was conducted using DNAMAN (version 6.0.3.99). A phylogenetic tree was constructed with MEGA 11.0 using the neighbor-joining method with 1000 bootstrap replicates. Conserved domains were identified via the Simple Modular Architecture Research Tool (SMART) (http://smart.embl-heidelberg.de/) (accessed on 30 October 2025). The predicted coding sequences of Amh and Amhr2 were obtained from the blotched snakehead genome (SRA Accession No. PRJNA730430) and the northern snakehead genome (SRA Accession No. PRJNA731586), respectively [42]. Three-dimensional protein structures were predicted via SWISS-MODEL (https://swissmodel.expasy.org/) (accessed on 30 October 2025), and molecular docking simulations were conducted using HDOCK (http://hdock.phys.hust.edu.cn/) (accessed on 30 October 2025), with results visualized in PyMOL (version 3.0.3).

### 4.3. Genomic Sequence Cloning and Structure Analysis

Genomic DNA was extracted from tail tissue using a Universal Genomic DNA Kit (CWBIO, Taizhou, China). The putative *Amhr2* genomic sequence was obtained from the blotched snakehead genome (SRA Accession No. PRJNA730430) [42]. Primers (Table 6) were designed to validate this sequence, and PCR products were cloned and sequenced as described in Section 4.2. The exon-intron structure was determined by aligning genomic and cDNA sequences following the GT/AG splice site rule.

For comparative analysis, *Amhr2* coding and genomic sequences from other teleosts were retrieved from the NCBI database (accession numbers listed in Table 7). Genomic structures were visualized using the Gene Structure Display Server 2.0 (http://gsds.cbi.pku.edu.cn) (accessed on 30 October 2025). Promoter core elements (PCE) and transcript start sites (TSS) were predicted with NNPP (http://www.fruitfly.org/seq_tools/promoter.html) (accessed on 30 October 2025). Putative transcription factor binding sites (TFBS) in the promoter region were predicted using AliBaba 2.1 (http://generegulation.com/pub/programs/alibaba2/index.html) (accessed on 30 October 2025) and Animal TFDB (https://guolab.wchscu.cn/AnimalTFDB4/) (accessed on 30 October 2025). Signal peptides and cleavage sites were predicted using SignalP-6.0 (https://services.healthtech.dtu.dk/services/SignalP-6.0/) (accessed on 30 October 2025), and subcellular localization with DeepLoc-2.1 (https://services.healthtech.dtu.dk/services/DeepLoc-2.1/) (accessed on 30 October 2025).

### 4.4. Estrogen Treatment

From 15 to 45 dpf, fry were fed for 30 days a diet supplemented with E_2_ (Macklin, Shanghai, China). The treatment window (15~45 dpf) was selected based on the critical period of gonadal sex differentiation in *C. maculata*, as demonstrated by Zhang et al. [25]. E_2_ was first dissolved in absolute ethanol, then evenly sprayed onto a commercial compound feed (Rongchuan, Guangzhou, China) and thoroughly mixed to ensure homogeneous distribution. The treated feed was air-dried in the shade to allow complete evaporation of ethanol before use. Based on our previous study [43], which reported a 70% sex reversal efficiency and the development of functional ovaries with this concentration, the experimental group received feed containing 30 mg E_2_ per kg. The control group was fed the same diet treated with ethanol only (0 mg/kg). Each treatment was conducted in triplicate tanks with equal stocking densities. During the experimental period, fish were reared in an open recirculating aquaculture system under controlled conditions: water temperature 26~28 °C, dissolved oxygen 6~8 mg/L, ammonia nitrogen < 0.5 mg/L, nitrite < 0.01 mg/L, and pH 7.0~7.5. After the treatment period, all fish were reared under the same standard conditions until sexual maturity.

At 105, 135, 165, and 195 days post-treatment (dpt), corresponding to 120, 150, 180, and 210 dpf, 30 fish were randomly sampled from each replicate of the E_2_-treated and control groups following MS-222 anesthesia. These four time points were selected based on the typical gonadal developmental cycle of *C. maculata* and the objective of assessing the stability of E_2_-induced sex reversal. In our previous study [43], gonads at 60~90 dpf were not fully reversed into ovaries. At 120 dpf, the ovaries of sex-reversed fish were essentially formed, and by 240 dpf, the ovaries contained abundant mature oocytes. The selected time points thus allowed us to determine whether the E_2_-induced gene expression pattern exhibits long-term stability. Using the sex-specific molecular marker and gonadal histology [22], individuals were classified as XY-M, XX-F, XY-I, and XY-F. Individuals were first classified into different sex categories using molecular markers and histological examination, and then randomly selected from each category (*n* = 3 per sex per time point) for expression analysis at the indicated dpt. Additionally, gonads from 105 dpt XX-F, XY-M, XY-I, and XY-F individuals were fixed in Bouin’s solution for HE staining and ISH.

### 4.5. Quantitative Real-Time PCR (qRT-PCR)

qRT-PCR primers are listed in Table 4, with *β-actin* as the reference gene, consistent with our previous study [44]. Reactions were performed in triplicate on a StepOnePlus™ Real-Time PCR System (ABI, Foster City, CA, USA) using SYBR^®^ Green Master Mix (Toyobo, Osaka, Japan) according to the manufacturer’s instructions. Each 20 μL reaction mixture contained 10 μL SYBR^®^ Green Master Mix, 0.8 μL each of forward and reverse primers, 7.4 μL ddH_2_O, and 1 μL cDNA. The thermal cycling protocol was as follows: 95 °C for 2 min; 39 cycles of 95 °C for 15 s, 50 °C for 15 s, and 72 °C for 30 s; followed by a melting curve acquisition step (60 °C for 5 s, then 95 °C for 5 s) using the instrument’s default settings. Relative expression levels of *Amhr2* were calculated using the 2^−∆∆Ct^ method. For tissue distribution, expression in the intestines (I) of XX-F individuals was set as the baseline (1.0). For gonadal development analysis, expression in the gonads of 30 dpf XX-F fish served as the baseline (1.0). In the estrogen treatment experiment, expression levels were normalized to those in 120 dpf XX-F individuals.

### 4.6. In Situ Hybridization (ISH)

Gonads from 120 dpf individuals were dissected, fixed in Bouin’s solution for 24 h at 4 °C, dehydrated, paraffin-embedded, and serially sectioned at 5 μm thickness. Consecutive sections were processed for HE staining and ISH following previously described procedures. For riboprobe synthesis, target gene fragments were amplified using gene-specific primers (Table 4). Digoxigenin (DIG)-labeled sense and antisense RNA probes were generated using a DIG RNA Labeling Kit (Roche, Mannheim, Germany) and purified via LiCl/ethanol precipitation.

Tissue sections were deparaffinized, rehydrated, and digested with 10 μg/mL proteinase K (CWBIO, Taizhou, China) at 37 °C for 15 min. Hybridization was performed with sense or antisense probes at 65 °C for 16 h. Signals were detected using an alkaline phosphatase-conjugated anti-DIG antibody (Roche, Mannheim, Germany; 1:1000 dilution) and visualized with NBT/BCIP (Roche, Mannheim, Germany). Sections were washed in 1×PBST, mounted with gelatin glycerin (Servicebio, Wuhan, China), and examined under a Nikon Eclipse Ti-U microscope (Tokyo, Japan).

### 4.7. Knockout of the Amhr2 Gene in C. maculata

CRISPR/Cas9 was used to generate *Amhr2* knockout mutants. Three guide RNAs (Amhr2-gRNA1, -gRNA2, and -gRNA3; Table 8) targeting the *Amhr2* gene were designed using CRISPRscan (https://www.crisprscan.org/) (accessed on 30 October 2025). The gRNAs were synthesized with a MAXIscript T7 In Vitro Transcription Kit (Invitrogen, Carlsbad, CA, USA) and purified via LiCl/ethanol precipitation. Prior to microinjection, each gRNA was mixed with TrueCut Cas9 Protein v2 (Invitrogen, Carlsbad, CA, USA). A mixture containing 200 pg gRNA and 300 pg Cas9 protein was co-injected into embryos at the 1~4 cell stage. Two control groups were included: 0.75% NaCl-injected and untreated. Each treatment was performed in triplicate with 300 embryos per replicate (900 embryos per group). Survival rates were assessed at 12 h post-fertilization (hpf; gastrula stage), 36 hpf (hatching stage), and 120 hpf (flat swimming stage). Five fry per group were collected at 48 hpf for genomic DNA extraction using a Universal Genomic DNA Kit. Given that F0 individuals are typically mosaic, early-stage screening using a small number of randomly selected embryos (commonly 5~10) is standard practice for confirming successful mutagenesis. Mutation detection used the primer pair Amhr2-JC-F/R (Table 4). Artificial insemination, microinjection, mutagenesis analysis, and fish rearing followed previously established protocols [41].

### 4.8. Statistical Analysis

All data are presented as mean ± standard deviation (SD). Statistical comparisons between groups were performed using Student’s *t*-test or one-way ANOVA followed by Dunnett’s post hoc test in SPSS version 26.0 (SPSS Inc., Chicago, IL, USA). Significance levels were set at *p* < 0.05, *p* < 0.01, and *p* < 0.001.

## 5. Conclusions

In this study, we successfully cloned and characterized the *Amhr2* ortholog in *C. maculata* and demonstrated its essential role in male sex differentiation and testis development. *CmAmhr2* exhibited male-biased expression in developing gonads and localized to germ cells, implicating it in early spermatogenesis. Its down-regulation following E_2_ treatment, concomitant with sex reversal, supports hormonal modulation of *CmAmhr2* expression. CRISPR/Cas9-mediated knockout confirmed that loss-of-function mutations disrupt the kinase domain critical for *Amhr2* function, providing a foundation for future mechanistic studies. These findings advance our understanding of sex differentiation pathways in *C. maculata* and establish a valuable molecular target for sex-controlled breeding in aquaculture.

## Figures and Tables

**Table 1:** Statistical summary of genetic sex ratios and gonadal phenotypes in fish exposed to varying E_2_ concentrations at 120 dpf (105 dpt).

E_2_ Concentration (mg/kg)	Genetic Sex		Gonadal Phenotype			Sex-Reversal Rate (%)
			Testis	Ovotestis	Ovary	
0 (Control)	XX	16	0	0	27	0.0
	XY	14	33	0	0	
30	XX	13	0	0	13	64.7
	XY	17	2	4	11	

**Table 2:** Fertilization, hatching, and survival rates of *C. maculata* embryos and fry in control and gRNA/Cas9-injected groups.

Group	Fertilized Eggs (N)	Embryos at Gastrula Stage (N)	Fertilization Rate (%)	Embryos at Hatching Stage (N)	Hatching Rate (%)	Fry at Flat Swimming Stage (N)
Amhr2-gRNA1	900	373 ^a^	41.4 ± 1.4% ^a^	311 ^a^	34.6 ± 2.0% ^a^	234 ^a^
Amhr2-gRNA2	900	384 ^a^	42.7 ± 2.0% ^a^	308 ^a^	34.2 ± 1.2% ^a^	211 ^a^
Amhr2-gRNA3	900	369 ^a^	41.0 ± 1.2% ^a^	303 ^a^	33.7 ± 1.5% ^a^	219 ^a^
0.75% NaCl	900	380 ^a^	42.3 ± 3.1% ^b^	312 ^a^	34.6 ± 1.1% ^b^	256 ^a^
Untreated control	900	726 ^b^	80.7 ± 2.0% ^b^	658 ^b^	73.1 ± 1.2% ^b^	577 ^b^

Note: Fertilization rate (%) = (number of embryos at gastrula stage)/(number of fertilized eggs) × 100%, hatching rate (%) = (number of embryos at hatching stage)/(number of fertilized eggs) × 100%. Values are presented as mean ± SD (*n* = 3). Different superscript letters within the same column indicate significant differences (*p* < 0.05), identical superscript letters indicate no significant difference (*p* > 0.05).

**Table 3:** Mutation types and efficiency of Amhr2 editing in F0 mosaic individuals.

Injected gRNA	Mutation Type	Number of Clone Carrying Mutated Amhr2	Mutation Rate
Amhr2-gRNA1	WT	15	50.0%
	−56	4	
	−45	3	
	−44	3	
	−26	2	
	−16	3	
Amhr2-gRNA2	WT	17	43.3%
	−3	3	
	−7	3	
	−13	2	
	+4	4	
	+6	1	
Amhr2-gRNA3	WT	18	40.0%
	−5	3	
	−8	4	
	−9	3	
	+2	1	
	+4	1	

Note: Deletions are denoted by “−” and insertions by “+”. Mutation rate (%) = (number of clones carrying mutated Amhr2)/(total examined clone) × 100%.

**Table 4:** Primers used for cDNA cloning, qRT-PCR, ISH, and knockout detection.

Primer Name	Sequences (5′-3′)	Application
Amhr2-F1	CCTAAGAGACGGTGTGCG	Partial sequence obtaining
Amhr2-R1	TCAAATAAATCAGAGCAGCG	
Amhr2-5′F-out	CACACCGTCTCTTAGG	5′-Race PCR amplification
Amhr2-5′R-in	ATCAGCCACCACAGTTGC	
Amhr2-3′F-out	GTGGAGGGTCATACTCAGATGGGCAC	3′-Race PCR amplification
Amhr2-3′R-in	GCAGGGAGACATCTATTCTTTGGGAC	
Amhr2-F2	GAGTTAACATGAACCTGC	ORF qualifying
Amhr2-R2	AATGTCAAGTCAACTGAA	
Amhr2-qF	GAGACGGTGTGCGTTCAAAG	qRT-PCR
Amhr2-qR	ATCGGGACAAGACTTTTCAACTA	
β-actin-qF	GCAAGCAGGAGTATGATGAG	
β-actin-qR	TTGGGATTGTTTCAGTCAGT	
Amhr2-ISH-F	TATGAGCTGCCACTGATGAA	In situ hybridization
Amhr2-ISH-R	CCAAAGAATAGATGTCTCCCT	
Amhr2-ISH-zy-F	TAATACGACTCACTATAGGGTATGAGCTGCCACTGATGAA	
Amhr2-ISH-zy-R	CCAAAGAATAGATGTCTCCCT	
Amhr2-ISH-fy-F	TATGAGCTGCCACTGATGAA	
Amhr2-ISH-fy-R	TAATACGACTCACTATAGGGCCAAAGAATAGATGTCTCCCT	
Amhr2-JC-F	ACAGGGACCGCTGAGAT	Knockout detection
Amhr2-JC-R	TAGAACATGGGTAAACACACAT	

**Table 5:** Accession numbers of Amhr2 amino acid sequences used for phylogenetic tree construction (NJ method).

Species	Gene	Accession Number	Species	Gene	Accession Number
*Anabas testudineus*	*Amhr2*	XP_026202805.1	*Oplegnathus punctatus*	*Amhr2cy*	[17]
*Bos taurus*	*Amhr2*	NP_001192257.1	*Oreochromis niloticus*	*Amhr2*	XP_003448346.2
*Channa argus*	*Amhr2*	XP_067361660.1	*Oryzias latipes*	*Amhr2*	ABF59994.1
*Channa maculata*	*Amhr2*	PZ213752	*Perca flavescens*	*Amhr2a*	XP_028432776.1
*Chelonia mydas*	*Amhr2*	XP_027683906.1	*Perca flavescens*	*Amhr2by*	XP_028443898.1
*Chrysemys picta*	*Amhr2*	XP_023965667.1	*Paralichthys olivaceus*	*Amhr2*	XP_019952149.1
*Cynoglossus semilaevis*	*Amhr2*	XP_024915829.1	*Phyllopteryx taeniolatus*	*Amhr2*	[12]
*Dicentrarchus labrax*	*Amhr2*	AGB07595.1	*Phyllopteryx taeniolatus*	*Amhr2y*	[12]
*Epinephelus coioides*	*Amhr2*	AXQ39882.1	*Plecoglossus altivelis*	*Amhr2*	BBP93678.1
*Gekko japonicus*	*Amhr2*	XP_015277694.1	*Plecoglossus altivelis*	*Amhr2-Y*	BBP93677.1
*Homo sapiens*	*Amhr2*	NP_065434.1	*Podarcis muralis*	*Amhr2*	XP_028577368.1
*Larimichthys crocea*	*Amhr2*	XP_027135385.1	*Scatophagus argus*	*Amhr2*	AYN77823.1
*Lates calcarifer*	*Amhr2*	XP_018534460.1	*Scophthalmus maximus*	*Amhr2*	XP_035487290.1
*Macaca mulatta*	*Amhr2*	XP_001105261.1	*Silurus meridionalis*	*Amhr2*	[13]
*Mastacembelus armatus*	*Amhr2*	XP_026162084.1	*Silurus meridionalis*	*Amhr2y*	[13]
*Monopterus albus*	*Amhr2*	XP_020445009.1	*Sturnus vulgaris*	*Amhr2*	XP_014748582.1
*Morone saxatilis*	*Amhr2*	XP_035517801.1	*Syngnathoides biaculeatus*	*Amhr2*	[12]
*Mus musculus*	*Amhr2*	NP_001343504.1	*Syngnathoides biaculeatus*	*Amhr2y*	[12]
*Numida meleagris*	*Amhr2*	XP_021238106.1	*Takifugu obscurus*	*Amhr2*	QGU34108.1
*Odontesthes hatcheri*	*Amhr2*	AWK67621.1	*Takifugu obscurus*	*Amhr2-Y*	QAT98470.1
*Oplegnathus punctatus*	*Amhr2a*	[17]	*Takifugu rubripes*	*Amhr2-X*	XP_011612373.2
*Oplegnathus punctatus*	*Amhr2ay*	[17]	*Takifugu rubripes*	*Amhr2-Y*	XP_011612373.2
*Oplegnathus punctatus*	*Amhr2by*	[17]	*Xenopus tropicalis*	*Amhr2*	XP_031753061.1

**Table 6:** Primers are used for genomic sequence cloning.

Primer Name	Sequences (5′-3′)	Length (bp)
Amhr2-gDNA-F1	TCTGTGTGTGACTAATGTGCCAA	1718
Amhr2-gDNA-R1	TCCCCTACAAGCCCACCAG	
Amhr2-gDNA-F2	GCTTCTCTCTTTTGTCTTCTGAT	1577
Amhr2-gDNA-R2	AAACCACATCTAAACAATCTGCT	
Amhr2-gDNA-F3	CAAAATACTGGAGATTACTGTGTGTG	1453
Amhr2-gDNA-R3	GATGACTCCTGACTTTGGTGTTC	
Amhr2-gDNA-F4	CCCAGATTGACCTCTCGTGTA	1110
Amhr2-gDNA-R4	TGCAGGTTCATGTTAACTCTAAGC	
Amhr2-gDNA-F5	TGGTCCATTTACAACTGCCT	1697
Amhr2-gDNA-R5	TTATCATTATTCTGACTTCTGTT	
Amhr2-gDNA-F6	CAGAAGTCAGAATAATGATAAGAAGA	1453
Amhr2-gDNA-R6	AGTGGCTTCCAATACTTCATCT	
Amhr2-gDNA-F7	CAACAGGAACATCTCTTGGACC	1419
Amhr2-gDNA-R7	CAGTCTTACTTGCTGTAGTTTGATGTC	
Amhr2-gDNA-F8	TTCACATTGCCCTCACTGATTTT	1135
Amhr2-gDNA-R8	AGGTTCATCACACTGCCATTA	
Amhr2-gDNA-F9	CTAATCATCCAGTCTATCCTAACG	837
Amhr2-gDNA-R9	GCCCATCTGAGTATGACCCTA	
Amhr2-gDNA-F10	GTCCTGTGTGATTTTGGATGCTC	1474
Amhr2-gDNA-R10	CTGAAAATGTGGTTTCATTTGTATTC	

**Table 7:** Accession numbers of genomic DNA (gDNA) and coding DNA sequence (CDS) for *Amhr2* orthologs in various species.

Species	Gene	CDS Accession Number	gDNA Accession Number
*Anabas testudineus*	*Amhr2*	XM_026347020.1	CM015722.1
*Channa argus*	*Amhr2*	XM_067505559.1	NC_090201.1
*Cynoglossus semilaevis*	*Amhr2*	XM_025060061.1	NC_024317.1
*Dicentrarchus labrax*	*Amhr2*	JQ801443.1	NW_026136711.1
*Lates calcarifer*	*Amhr2*	XM_018678944.2	NC_066844.1
*Monopterus albus*	*Amhr2*	XM_020589353.1	NW_018127881.1
*Morone saxatilis*	*Amhr2*	XM_035661908.1	NW_023339740.1
*Paralichthys olivaceus*	*Amhr2*	XM_020096590.2	NC_091094.1
*Perca fluviatilis*	*Amhr2a*	XM_028576975.1	NC_041334.1
*Perca fluviatilis*	*Amhr2by*	XM_028588097.1	NC_041339.1
*Scatophagus argus*	*Amhr2*	MH238356.1	NC_058495.1

**Table 8:** gRNA and primer sequences for targeting *Amhr2* in *C. maculata*.

Primer Name	Sequences (5′-3′)	Locus
Amhr2-gRNA1	TAATACGACTCACTATAGGAATACCAGGGATCCATAG**GTTTTAGAGCTAGAAATAGC**	Exon 5
Amhr2-gRNA2	TAATACGACTCACTATAGAGAATGGAAGCATATATTTA**GTTTTAGAGCTAGAAATAGC**	Exon 5
Amhr2-gRNA3	TAATACGACTCACTATAGCTGGGATTGTCAACTTCCTG**GTTTTAGAGCTAGAAATAGC**	Exon 5
Common reverse	AAAAAAAGCACCGACTCGGT	/

Note: Target sites are double-underlined, and bold text denotes the plasmid scaffold sequence.

## Data Availability

All the data related to this project is available from the corresponding author and will be provided on request.

## Supplementary Materials

The following supporting information can be downloaded at: https://www.mdpi.com/article/10.3390/ijms27114884/s1.
