# Supplementary material for: Molecular Characterization and Functional Analysis of Amhr2 in Sex Differentiation and Gonadal Development of Blotched Snakehead (Channa maculata)
Source: Int J Mol Sci. 2026 May 28;27(11):4884. doi: 10.3390/ijms27114884 (PMC13256886; doi:10.3390/ijms27114884)
Supplement: Supplementary file 1 [file ijms-27-04884-s001.zip › Supplementary Figure 1-6/Supplementary Figure 1.pdf]

1 ggttcagtgtcttgcccagaaacactttgacatatagctgggaccgggggtcgaaccactgaccctgtggtccatggacaactgccttac

91 caactgagctgcagatgaatttatcaataaatgttgtgtattctcacagcccattgtttggcagatggtatgtgtagtacacgcaggtgt

181 gtgggagggatgctgggaaatggtaaactttttttcacattcccaggactgataaaaactcccacctgactccagtctaggcttagagtta

1 M N L Q L W W L I L S V E C I F V C I Y S Q S L P K R R C A

271 ac**ATG**AACCTGCAACTGTGGTGGCTGATTTTGTCTGTGGAATGCATCTTTGTATGTATCTACAGCCAGTCTTTACCTAAGAGACGGTGTG

31 F K V T A Q N N K Y T I A G K V N E S V Q V C E N T Q C C V

361 CGTTCAAAGTGACAGCGCAGAACAACAAGTACACAATAGCTGGAAAGGTGAATGAGTCTGTGCAGGTCTGTGAAAACACCCAGTGCTGTG

61 S Y Y V V I N G Q P K V D V L A C D I V E K S C P D A T C K

451 TGAGTTATTACGTGGTCATCAATGGCCAGCCGAAGGTTGACGTTCTCGCTTGTGATATAGTTGAAAAGTCTTGTCCCGATGCAACCTGCA

91 A E T R F N G R L I K C V C N T D L C N R N I S W T T G S E

541 AGGCAGAAACACGCTTTAATGGTCGCCTCATTAAATGCGTGTGCAACACAGACCTCTGCAACAGGAACATCTCTTGGACCACAGGATCTG

121 Q L Q L T Y S Y S K G T A E I E I D D I K L Q Q I L H R G H

631 AACAGCTTCAACTCACCTACTCTTATTCTAAGGGGACCGCTGAGATTGAAATTGATGACATCAAACTACAGCAAATTTTACACCGTGGGC

151 F A T V W Q G E Y Q G S I V A V K V F P A E W K H I F T A E

721 ATTTTGCAACTGTTTGGCAAGGGGAATACCAGGGATCCATAGTGGCTGTGAAGGTTTTCCCAGCAGAATGGAAGCATATATTTACGGCAG

181 K E V Y E L P L M K H A G I V N F L G T G K E P D C S S W L

811 AGAAAGAGGTTTATGAGCTGCCACTGATGAAGCATGCTGGGATTGTCAACTTCCTGGGGACTGGGAAGGAACCAGATTGTAGCAGCTGGC

211 I V L Q L A E Y G S L H S Y L R K H T T N W T L S L K F C Q

901 TTATTGTCCTGCAACTTGCTGAATATGGTTCTCTTCATTCCTATTTGCGTAAACACACCACCAACTGGACGTTGTCACTGAAGTTTTGCC

241 S L S E G L S F L H S D L H K Y D V H K P S V A H R H L S S

991 AGTCTTTATCAGAGGGACTTTCCTTTCTTCACTCTGACCTTCACAAATATGATGTGCATAAACCTTCTGTGGCTCACAGACACCTCAGCA

271 S N V L V R A D D S C V L C D F G C S T I L R S C S G Q W L

1081 GCTCCAATGTGCTTGTGAGAGCAGATGATAGCTGTGTCCTGTGTGATTTTGGATGCTCCACCATCCTGCGTTCCTGTTCAGGACAATGGC

301 S P M T S V E G H T Q M G T L R Y M S P E I L E G S V N L S

1171 TGAGCCCAATGACAAGCGTGGAGGGTCATACTCAGATGGGCACACTGCGCTACATGTCCCCTGAGATCCTGGAAGGCTCTGTAAACCTAA

331 S S S C L M Q G D I Y S L G L L L W E I W M R C S D L F E G

1261 GTAGCAGTTCATGTCTCATGCAGGGAGACATCTATTCTTTGGGACTGCTACTGTGGGAGATCTGGATGCGCTGCTCTGATTTATTTGAAG

361 G I V P P H L L P Y E S E L G A D P M L K D L L Q F V F Q M

1351 GCGGCATTGTTCCTCCACATCTTCTGCCTTATGAATCCGAGCTGGGAGCTGATCCAATGTTGAAAGATCTCCTCCAGTTTGTATTTCAAA

391 Q K R P S I P E H W A L L P Q G S A L Q E L L T D C W D W D

1441 TGCAAAAGAGACCATCCATACCTGAACACTGGGCGTTGCTACCACAGGGATCAGCACTGCAGGAGCTCCTGACAGATTGTTGGGACTGGG

421 P D A R L T T Q C V L E R L A S L Q P C Y S P *

1531 ACCCTGATGCCCGACTGACAACTCAGTGTGTTTTGGAGAGATTAGCCTCTCTTCAGCCTTGTTATTCTCCA**TGA**catttttttttcagtt

1621 gacttgacattttttgcattcaagcatttacacagttttttttagtatatttacattgtgttgaactgtgaagatctgtaggttacttgt

1711 tcccattacagtctgtctttccatttaatgcttaaaatgttgttttaaaaatgtaaaaaaaatgtaaatgctggattaaaaatgtaatcc

1801 attagcattatagaaaacttgaaaagcatgtccataatgccataatgcaacaaattgtgggaatacaaatgaaaccacattttcagtaaa

1891 aaaaaaaaaaaaaaaaaaaaaaa
