# Supplementary material for: Molecular Characterization and Functional Analysis of Amhr2 in Sex Differentiation and Gonadal Development of Blotched Snakehead (Channa maculata)
Source: Int J Mol Sci. 2026 May 28;27(11):4884. doi: 10.3390/ijms27114884 (PMC13256886; doi:10.3390/ijms27114884)
Supplement: Supplementary file 1 [file ijms-27-04884-s001.zip › Supplementary Figure 1-6/Supplementary Figure 2.pdf]

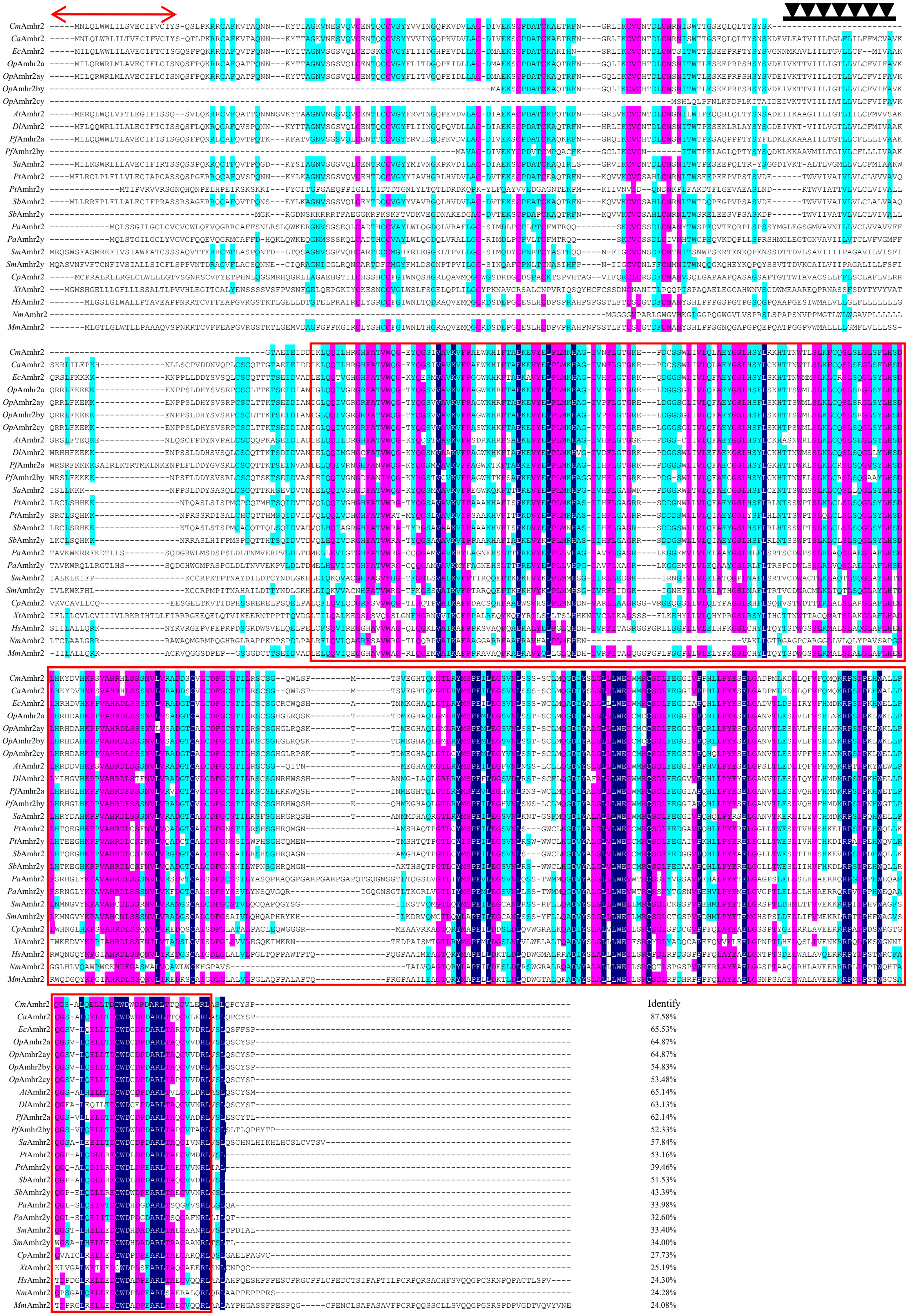
CmAmhr2
CaAmhr2
EcAmhr2
OpAmhr2a
OpAmhr2ay
OpAmhr2by
OpAmhr2cy
AtAmhr2
DlAmhr2
PfAmhr2a
PfAmhr2by
SaAmhr2
PtAmhr2
PtAmhr2y
SbAmhr2
SbAmhr2y
PaAmhr2
PaAmhr2y
SmAmhr2
SmAmhr2y
CpAmhr2
XtAmhr2
HsAmhr2
NmAmhr2
MmAmhr2
Identify
87.58%
65.53%
64.87%
64.87%
54.83%
53.48%
65.14%
63.13%
62.14%
52.33%
57.84%
53.16%
39.46%
51.53%
43.39%
33.98%
32.60%
33.40%
34.00%
27.73%
25.19%
24.30%
24.28%
24.08%
