# Supplementary material for: Molecular Characterization and Functional Analysis of Amhr2 in Sex Differentiation and Gonadal Development of Blotched Snakehead (Channa maculata)
Source: Int J Mol Sci. 2026 May 28;27(11):4884. doi: 10.3390/ijms27114884 (PMC13256886; doi:10.3390/ijms27114884)
Supplement: Supplementary file 1 [file ijms-27-04884-s001.zip › Supplementary Figure 1-6/Supplementary Figure 5.pdf]

```
-1800 caatttgaattctgatggatataagaagcaaagattgttcacttggctggttacaactctcaatgttaaaccacaattttcacataatgttttgatctcataaatctaaatgtaaataat
-1680 taaaccatgatttggtttggatttgaaaagtgcatctacaggtcttgaactctttattccagcaactattatacctaaagctaagacttttgtttccccgttggtatgtaacgctgttt
-1560 gtggagtcgtggagcccacagccgaaaaaaaaaatcccaccttgtcgctgagttcactttcagaaaagccataaaacgtggagtcattgtcagagtcctccgcgaaaatatcagctaact
-1440 cctttgagagagtgtgcattccccaaacaccagtctgaagaaagatgcatcaacaaaattagtgaaaaactgttttaacctgtatcttaacaaatgtgtaaaactgagttactgtaccat

-1320 tttattagagggtagcacgggttgtaggtgacttctagcttgtaaagattacgcgcaggcacacgggcctaagatttcagtcaaattcagatgcgcttgatttgtacgtaagggcagcgt
      AR
-1200 aaatctacagaactgccctggaatcatgttttattttgatctccgcccccggaggttgtagcgaagaatcacacgtgcggccagttttctgatacattctgattattgtagattattc
      SP1  WT1  DMRT1
-1080 gattaaagggtgtttcccagacaagatttccaaaatctcaaacacttaatttgtattcagcatggcaagtttgagaaagagaaaaaagtgggttaaggttaatgggttaaagggataaaa
      Smad2  C/EBP  GATA-1  GR
-960  cccagattgacctctcgtgtattaacattacaggacaggctaagcatgtttagcatgttagcatgctaacaaaacacaaattacagcttgaagcagatcgggatgtcaacatctattcac
      C/EBPalp
-840  atagttattcacaaactaaagtgcacatgcaaagtttgatcaggtgatgcataatcaagtcagtgaacaccaaagtcaggagtcatcctttggggtccatgaatatacagtacaaaacac
      AP-1  Esr1  SOX9
-600  ttatggcaatccatccaatattagttgagatatttctcaatggaatggtgcaatggccaatggcaaaaaatctaatgtattccatcatttgaagatgctcatgatttgaggtaagttttt
      C/EBP  AP-1
-720  caaaggtttttacttcatgtatacaaattaaggcctagaatatgatacttttgagataccatgagtatatttattaccgtaatttctaacatgcaatttcttcttagtgtaaatcatcaa
      Oct-1  C/EBPalp
-600  aaagaaattcatttctcatttattcaaataaaatgtatacaaataaagtgcttagagagatgttttctgaaaggtggggtcttaatatactaagacagctgcaaaaaaacactccact
      Foxl2  Myod
-480  tcaatcagacaaaattcctaaacagtgtgagattttttttttttttttttcttttcgtccttttggcttatcccatgagttcagggtcgccacagtggatcattgtctgcattttggcct
      GR  SOX9
-360  agtttttttttttttttacgccggatgcccctcctgacacaaccctccccaatttctaccaggcttgaacgggcactgcactgctggggatgggaaaggctgttgggggttcagtgtctt
      SP1
-240  gcccagaaacactttgacatatagctgggaccggggatcgaaccactgaccctgtggtccatttacaactgccttaccaacttagctgcagatgaatttgtcaataaatgttgtgtattc
      GATA-1
-120  tcacagttggcagatggtatgtgtagtacacgcaggtgtgtgggagggatgctgggaaatggtaaactttttttcacattcccaggactgataaaaactcccacctgactccagtctaggc
      Smad3  Smad3
+1    ttagagttaacATGAACCTGCAACTGTGGTGGCTGATTTTGTCTGTGGgtgagtgtaacccattttaatgctctttaggtttgtgtttttttctttagttttccttagttttttttacatg
      TSS  TIS
```
