# Supplementary figures and images for: Molecular Characterization and Functional Analysis of Amhr2 in Sex Differentiation and Gonadal Development of Blotched Snakehead (Channa maculata)
Source: Int J Mol Sci. 2026 May 28;27(11):4884. doi: 10.3390/ijms27114884 (PMC13256886; doi:10.3390/ijms27114884)

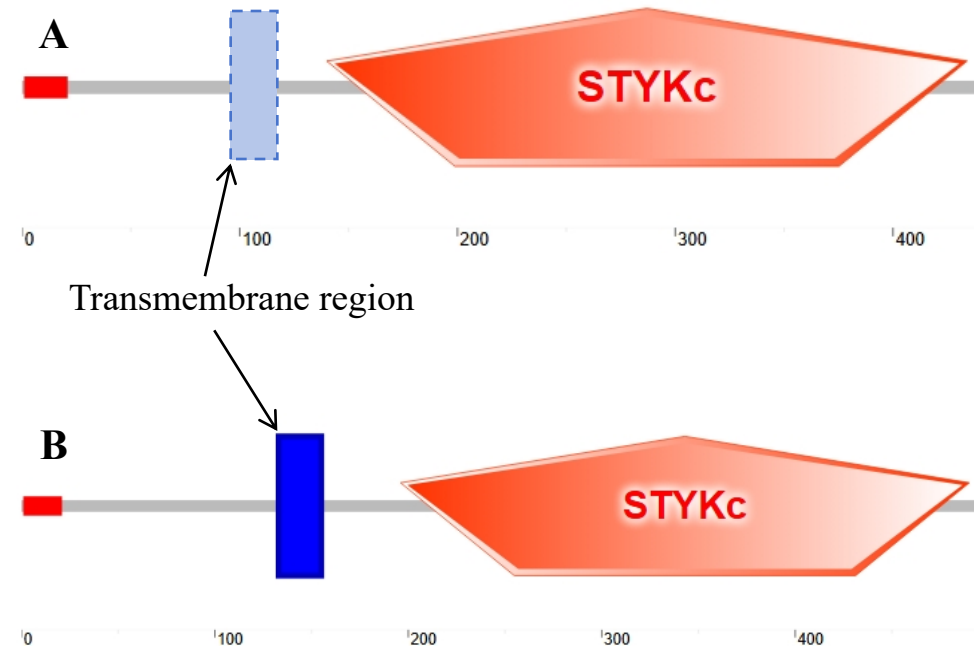

Supplement: Supplementary file 1 [file ijms-27-04884-s001.zip › Supplementary Figure 1-6/Supplementary Figure 3.pdf]

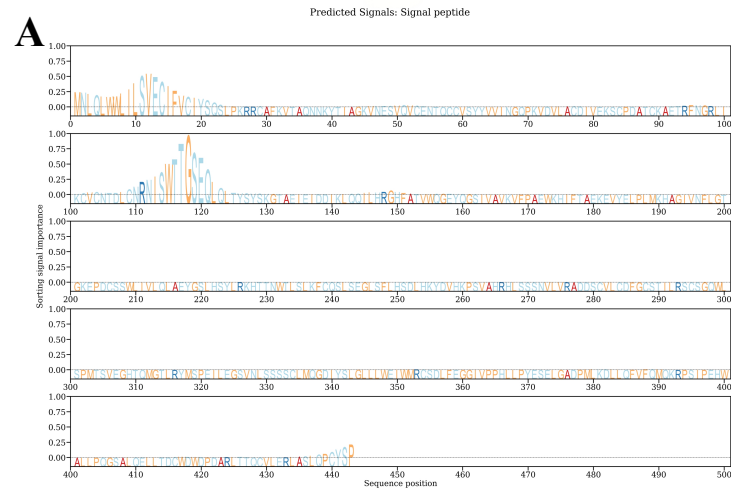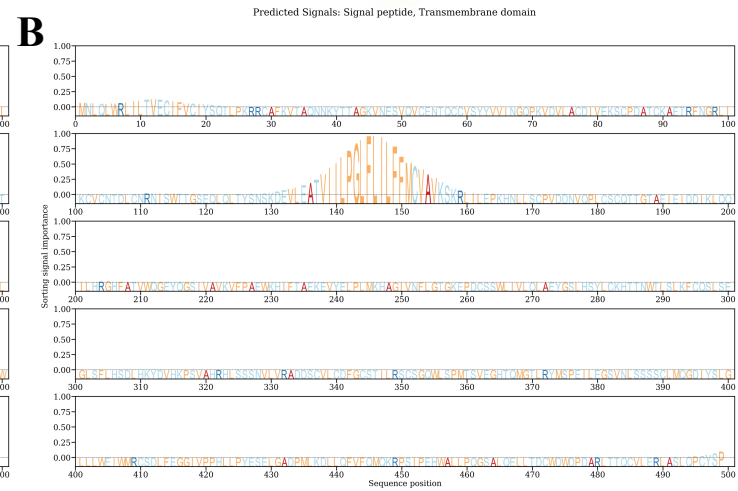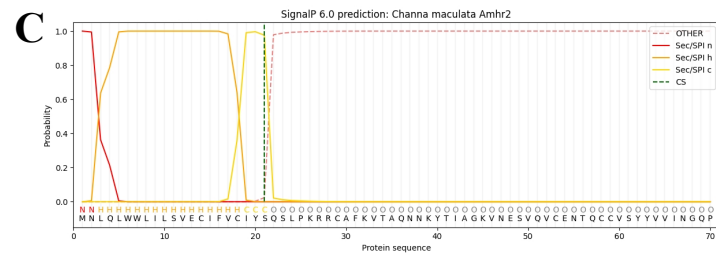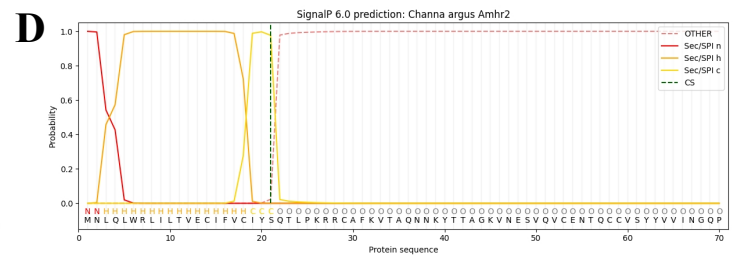

Supplement: Supplementary file 1 [file ijms-27-04884-s001.zip › Supplementary Figure 1-6/Supplementary Figure 4.pdf]

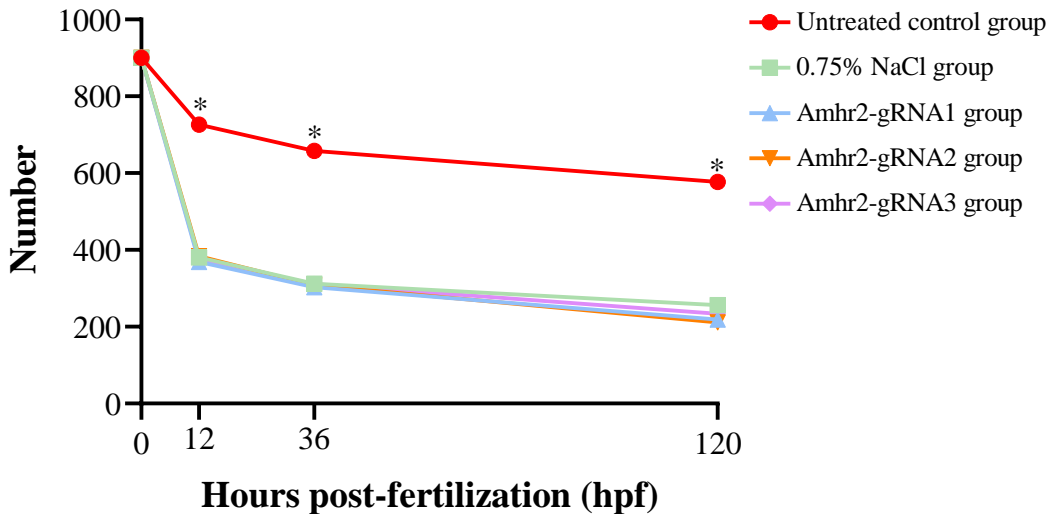

Supplement: Supplementary file 1 [file ijms-27-04884-s001.zip › Supplementary Figure 1-6/Supplementary Figure 6.pdf]
